# Anthrax in Humans, Animals, and the Environment and the One Health Strategies for Anthrax Control

**DOI:** 10.3390/pathogens13090773

**Published:** 2024-09-07

**Authors:** Deepak Subedi, Saurav Pantha, Sumit Jyoti, Bickal Gautam, Krishna Kaphle, Rakesh Kumar Yadav, Shristi Ghimire, Santosh Dhakal

**Affiliations:** 1 Paklihawa Campus, Institute of Agriculture and Animal Science (IAAS), Tribhuvan University, Siddarthanagar 32900, Nepal; sauravvet@gmail.com (S.P.); sujy12@gmail.com (S.J.); thevetbickal@gmail.com (B.G.); krishnakaphledr@gmail.com (K.K.); rakeshy792@gmail.com (R.K.Y.); 2 Department of Poultry Science, University of Georgia, Athens, GA 30602, USA; 3 Department of Diagnostic Medicine/Pathobiology, College of Veterinary Medicine, Kansas State University, 1800 Denison Avenue, Manhattan, KS 66506, USA; sghimire1@vet.k-state.edu; 4 Department of Health Management, Atlantic Veterinary College, University of Prince Edward Island, 550 University Avenue, Charlottetown, PE C1A 4P3, Canada; 5 Department of Pharmacology, Dalian Medical University, Dalian 116041, China

**Keywords:** anthrax, *Bacillus anthracis*, biological weapon, spores, One Health approach

## Abstract

Anthrax is a notorious disease of public health importance caused by *Bacillus anthracis*. The causative agent can also be used as a biological weapon. Spores of these bacteria can sustain extreme environmental conditions and remain viable in soil for decades. Domestic and wild ruminants are highly susceptible to this pathogen, which usually presents as a peracute to acute disease. In humans, cutaneous anthrax is frequent but pulmonary and enteric anthrax are more serious. Humans, animals, and the environment are all involved, making anthrax a perfect target for a One Health approach. The environment plays a key role in disease transmission. At a time when the One Health concept is not mere slogans, collaborative efforts of medical professionals, veterinarians, and environmental scientists will be valuable for the prevention and control of this disease. In this review, we discussed the transmission dynamics of anthrax in the environment, animals, and humans, as well as One Health strategies to control and prevent anthrax.

## 1. Introduction

**Figure 1:**
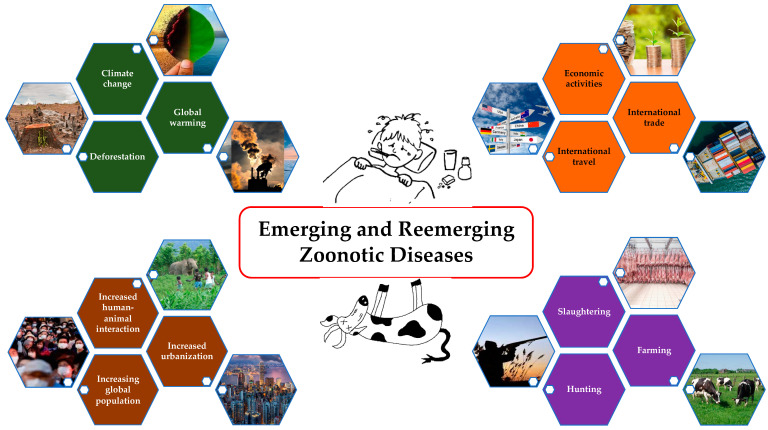
Causes of emerging and remerging zoonotic diseases.

Rising global population; increased urbanization and human–animal interactions; severe ecological changes like deforestation, global warming, and climate change; changes in socio-cultural behaviors such as hunting, farming, and slaughtering; and increased international trade, travel, and economic activities have altogether resulted in the emergence and reemergence of the zoonotic diseases (Figure 1) [1,2,3,4]. Approximately 75% of new diseases that have infected humans in the last decade are caused by pathogens of animal origin [5]. Anthrax was identified as a priority by 65 countries; however, only one burden of disease (BoD) study has been conducted [6]. Notably, anthrax is absent from both the Global Burden of Disease (GBD) study and the WHO roadmap, even though it is the only bacterial zoonotic disease included in the World Health Assembly resolution WHA 66.12 on neglected tropical diseases (NTDs) [7]. People who heavily rely on domestic and wild animal life for their subsistence, who live close to these species, and who lack adequate information about disease spillover and sanitation are more likely to suffer from these diseases [8]. These challenges make it difficult to gain support and advocacy for addressing neglected zoonotic diseases unless there is a major pandemic threat that draws attention to these issues [9]. As a result, these diseases straddle the threshold between animal health and human health. Given that disease control depends on the cooperation of stakeholders from human, animal, and environmental sectors as well as the rest of the world to achieve success, the “One Health” (OH) concept needs to bridge the gap.

The notion of OH is a multi-sectoral strategy to reduce zoonotic infections in people and animals [10,11]. It is considered today the most appropriate approach for collaborative, multi-sectoral, sustainable, and realistic initiatives to attain optimal health for humans, animals, and the environment [10,12]. A wide range of environmental issues can be alleviated by the implementation of OH measures. These include checks on environmental contamination, habitat conflicts, biodiversity loss, the development of infectious illnesses, antibiotic resistance, and ecosystem function deterioration. Since the recent COVID-19 pandemic, OH has proven to be a successful strategy for addressing a wide range of issues from vaccine research to mass awareness [13,14].

Anthrax is a bacterial archetype zoonosis caused by *Bacillus anthracis*, which has a wide range of susceptible hosts including humans [15]. It is one of the major globally neglected diseases, which is both zoonotic and endemic [16]. People are most likely to become infected when handling animal carcasses, skins, hair, and bones infected with *B. anthracis*. Over the last hundred years, there have been numerous documented anthrax outbreaks due to both natural and intentional causes, globally [17,18,19,20,21,22]. There are an estimated 1.8 billion people at risk of anthrax transmission, including more than 60 million livestock owners and 1.1 billion animals [23,24]. This review aims to summarize anthrax prevalence in humans, animals, and the environment and present the ideas of the OH approach that may strengthen and support the ongoing initiatives to prevent anthrax.

## 2. Transmission Cycle of Anthrax

**Figure 2:**
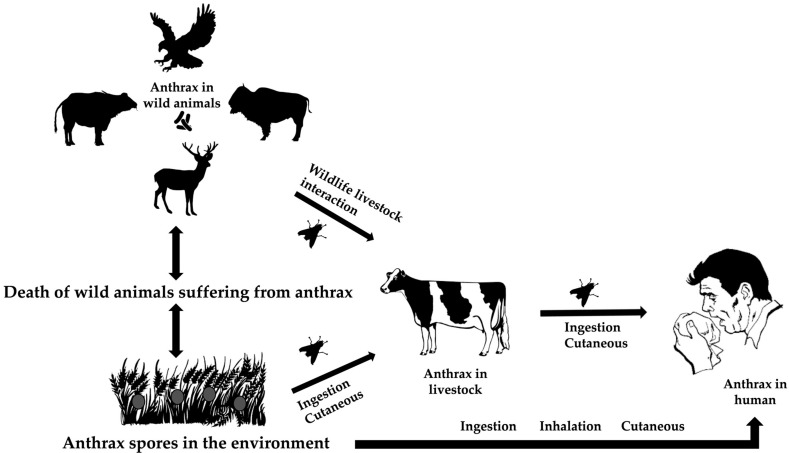
The life cycle of *Bacillus anthracis* at the animal–environment–human interfaces.

Ecological factors at the intersection of wildlife, livestock, and the environment drive the bacterial spread. Due to the high level of virulence of *Bacillus anthracis,* it usually kills its hosts throughout its life cycle [25]. The spores of the bacterium may live for years in harsh environments, such as high temperatures, UV and ionizing radiation, chemicals, and pressure [26]. Ingestion, cutaneous transmission through damaged skin, or inhalation while grazing are different ways that herbivores and animals are exposed to resistant spores. The susceptible species become infected, die, and release the spore back into the environment [27] (Figure 2).

Ruminants and primates, including humans, are more susceptible to the disease than other species [18]. Other animals affected by the disease include zebras, rhinoceroses, elephants, and ostriches. It is widely accepted that sporulated forms released from these carcasses are the primary source of infection to the healthy susceptible hosts through grazing pastures, drinking contaminated water, or other means [28,29].

In some enzootic zones, flies, such as *Tabanus*, *Stomoxys*, and *Chrysomia*, are considered suspects for modifying the spread of anthrax and causing outbreaks [30,31]. Blackburn et al. (2014) provided empirical and genetic evidence showing that flies can carry *B. anthracis* from infected animal carcasses to nearby vegetation through their emesis and feces [32]. This contamination of vegetation can then potentially infect other herbivores that browse on these plants, thereby expanding the spread of anthrax in wildlife [32].

The interaction between wildlife species and livestock (wildlife–animal interface) is directly or indirectly recognized as a potential means of inter-species animal transmission in a given landscape, and subsequent spillover to the human population [27]. The introduction of the infected livestock to the wild herbivores can cause sporadic transmission [33].

Humans can be infected by handling contaminated items such as carcasses, meat, skins, hair, and bones (industrial anthrax) as well as by handling animals during treatment, transport, or disposal of their carcasses (non-industrial anthrax) [29]. Humans are dead-end hosts and inter-human transmission of the disease has not yet been reported [34]. Disorientation, ataxia, respiratory distress, and apoplectic seizures are some of the clinical signs seen in ruminants when anthrax is present as a peracute or acute disease. Acute death frequently occurs with bloody discharge from natural orifices, bloating, an incomplete or absent rigor mortis, and absence of blood clotting [25]. Approximately 95% of all human cases of anthrax are caused by cutaneous anthrax, with inhalation (Woolsorter’s disease) and gastrointestinal anthrax accounting for the remaining occurrences [35].

## 3. Anthrax in the Environment

The bacterium exists in two forms: vegetative form and spore form. The spores are resistant to desiccation from the harsh environmental conditions and these spores must enter the susceptible hosts through a viable route to germinate and form vegetative forms capable of propagating and eventually killing the hosts [26]. Sporulation is a resting stage and not a form of replication, and those spores, as mentioned above, are resistant to prolonged desiccation, extreme pH, temperature, pressure, and ultraviolet radiations. Thus, the spores can persist in the environment for an extended period [36]. Sporulation, the process by which bacteria form spores, is dependent on oxygen. Adequate oxygen levels are necessary, particularly in the early stages, to ensure successful spore formation [37]. A recent study by Hsieh et al. (2023) found that the *B. anthracis* Sterne strain could sporulate under anaerobic conditions, but with varying efficiency depending on the medium. Solid media, like sheep blood agar plates, showed a significant 4–5 log reduction in spore titers, while liquid blood cultures had a smaller reduction. When cultures were initially incubated with oxygen and then moved to anaerobic conditions, spore titers remained similar to those in fully aerobic environments. The sharp decline in sporulation on solid media under anaerobic conditions was not solely due to reduced bacterial growth, indicating other factors at play [38].

Certain microenvironments favor bacterial sporulation and thus, the incidence of anthrax is higher. Examples include areas with low depressions where standing water could collect and devitalize the plant life or rock lands, and the hillside seeps, which could accumulate organic matter during the water runoff [26]. The occurrence of anthrax in those areas is stimulated by the presence of limestone but hindered by the presence of sandstones [39]. The incidences of anthrax outbreaks are higher in the dry summer months [40] that follow prolonged periods of rain and in the presence of calcareous soils (i.e., with high calcium levels and consequently abundant in nutrients [41]). The cycles of spore germination, vegetative outgrowth, and re-sporulation are supported by alkaline pH, high soil moisture, and the presence of large quantities of organic matter [26,39]. Driciru et al. (2020) predicted that hot–dry climatic conditions, combined with alkaline soils rich in potassium and calcium ions, act as key ecological drivers that facilitate the survival of *B. anthracis* spores and trigger subsequent anthrax outbreaks [42].

The role of animals in the transportation of the spores in the environment has also been discussed. Carnivores are less susceptible to anthrax; therefore, they can ingest spores without showing any clinical indications, and they act as carriers by shedding spores in their feces into the environment. Birds such as raptors, herring gulls, and vultures all play a role in the long-distance spread of spores [43,44,45]. Vector insects like tabanids and mosquitoes are implicated in the transmission of spores from animals to vegetation [30,31]. Likewise, the soils that adhere to the coat of wallowing bison in the anthrax-prone areas [26], water, and wind can also disperse the spores in the environment [46].

## 4. Animal Anthrax

Outbreaks of anthrax are observed in various animal species. Most of the domesticated herbivores are extremely sensitive while carcass scavengers are generally immune to anthrax [25]. Cormack et al. (2001) reported that 23 free-ranging bovid species, six cervid species, 17 carnivore species, and two primate species were confirmed to have died of anthrax worldwide, along with several free-ranging zebra and rhino species, and Asian and African elephants [47]. Sporadic outbreaks of anthrax cause the loss of livestock and wildlife alike in endemic locations [48]. Meat and bone meal salvaged from stagnating stock in affluent countries acts as the primary route of transmission, whereas transporting animals in cargo shipments can enhance the potential for cross-contamination [23].

Naturally infected animals have an incubation period of 1 to 14 days or more [18]. In bovine species, anthrax usually develops as an acute febrile disease, and clinical signs include an abrupt rise in fever, and irritability followed by dullness and death of animals within 2–3 days if no treatment is offered. Shivering, cramp-like signs, blood-stained urine, and respiratory distress are also observed in infected cattle species [49,50]. In animals infected with *B anthracis*, bloody discharge from natural orifices, bloating, incomplete or absent rigor mortis, and absence of blood clotting are (generally but not always) observed. Anthrax normally manifests as a single acute disease in sheep and goats but may differ in immunized animals [51]. When infected with *B. anthracis*, pigs and other carnivores have local edema, especially around the neck [29]. Dogs and cats are usually quite resistant to anthrax [52]. While cases and deaths are observed in ostriches [53], anthrax is rare and susceptibility is low in birds [54,55]. There are reports of wild mammal deaths in national parks around the world, with anthrax being the most common cause of death in southern and central Africa [25]. Carnivores are more resistant to anthrax than herbivores, yet they can still succumb to the disease. If animals are not treated, the blood of most susceptible species contains 10^7^ to 10^9^ bacilli per milliliter at the time of death. Pigs often have a lower quantity of bacteria than the other livestock species for reasons that are unknown [23]. Inhalation, ingestion, or cutaneous contact, through the broken skin or mucous membrane, with a spore may cause infection in animals and humans [18]. The 50% lethal dose (LD_50_) by oral route in herbivores is estimated to be around 10^7^ to 10^8^ spores administered as a single dose [56,57].

Anthrax is present globally on all continents except Antarctica. Certain regions experience more frequent outbreaks, while others encounter sporadic outbreaks triggered by unusual weather patterns. These conditions can bring dormant spores in soil to the surface, where they are ingested by ruminants, germinate, and cause illness [58]. In several states of India, including Andhra Pradesh, Jammu and Kashmir, Tamil Nadu, Odisha, and Karnataka, anthrax is enzootic. In Bangladesh, a total of 6354 animal anthrax cases were reported, resulting in 998 deaths, with an overall case fatality rate of 15.7% from 1980 to 2023 [59]. In Nepal, approximately forty livestock died in the Palpa district in 2016. In 2019, at least 44 cows were found dead about 700 m from the Koshi Tappu Wildlife Reserve in the Sunsari district of Nepal. The disease was later identified as anthrax [60].

In 2019, Texas in the USA experienced a surge in anthrax-positive cases across various animal species, including cattle, white-tailed deer, goats, horses, and exotic antelope [61]. In 2022, an anthrax outbreak in Sierra Leone resulted in the deaths of 233 animals [62] and 29 laboratory-confirmed animal anthrax cases were reported in Croatia [20]. In 2023, a total of 35 anthrax cases (20 cattle and 15 sheep) were recorded, resulting in 23 deaths (13 cattle and 10 sheep) in Nigeria [22]. In Australia, anthrax occurs in the “anthrax belt”, which runs from western New South Wales into part of Victoria [63]. Anthrax has caused significant production losses in both domestic and wild animals since ancient times. However, its incidence significantly decreased after the vaccine was discovered. In the past three decades, the disease has become sporadic in most regions where regular livestock vaccination programs are in place [52].

## 5. Human Anthrax

**Figure 3:**
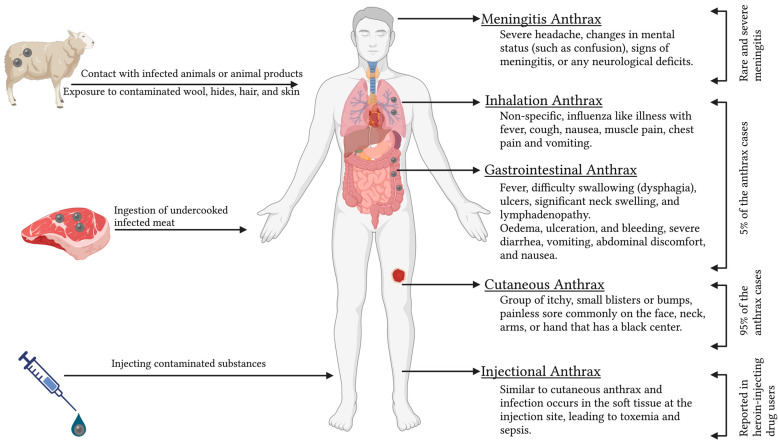
Different forms of human anthrax.

Human anthrax is caused either by contact with infected animals or animal products; ingestion of undercooked infected meat; exposure to contaminated wool, hides, hair, and skin; or by injecting contaminated substances [64]. Anthrax in humans can manifest in cutaneous, respiratory, and gastrointestinal forms, with 95% of the patients showing cutaneous symptoms and 5% of patients with inhalation or gastrointestinal syndromes (Figure 3) [24]. All four types of anthrax (cutaneous, gastrointestinal, inhalation, and injectional) in humans can be lethal if left untreated, but the cutaneous form is more typically self-limiting [24].

### 5.1. Cutaneous Anthrax

The incubation period typically lasts from 2 to 7 days, with a possible range of 1 to 19 days [65]. Cutaneous anthrax is distinguished by pruritic papules that develop into vesicles, as well as regional edema that is frequently severe and affects a vast anatomical area [66]. Cutaneous lesions heal to form a painless eschar with a dark necrotic core [35]. Uncomplicated cutaneous anthrax heals with minimal scarring with antibiotic treatment, but around 20% of untreated cases evolve into systemic infection, which can be fatal [67]. When diagnosing cutaneous anthrax, it is important to consider other conditions such as cutaneous tuberculosis, ecthyma gangrenosum, erysipelas, glanders, orf, plague, rat-bite fever, rickettsial infection, staphylococcal and streptococcal skin and lymph node infections, syphilitic chancre, and ulceroglandular tularemia [65,68].

### 5.2. Gastrointestinal Anthrax

Infection occurs 3 to 7 days after ingesting *B. anthracis* through contaminated food or drinks [65,69]. Lesions can develop at any point along the gastrointestinal tract. There are two clinical forms: oropharyngeal and gastrointestinal. Oropharyngeal anthrax is characterized by fever, difficulty swallowing (dysphagia), ulcers, significant neck swelling, and lymphadenopathy [70]. Gastrointestinal anthrax causes oedema, ulceration, and bleeding, resulting in symptoms of severe diarrhea, vomiting, abdominal discomfort, and nausea [25]. Lesions most commonly develop on the wall of the terminal ileum or cecum [65].

### 5.3. Inhalation Anthrax

An occupational disease of “Woolsorters” is pulmonary anthrax as a consequence of inhalation of bacterial spores from contaminated wool and hair that causes non-specific, influenza-like illness with fever, cough, nausea, muscle pain, chest pain, and vomiting [71]. Septicemia and meningitis are frequently caused by inhalation anthrax, which has the highest fatality rate and the greatest chance of human-made dissemination [72]. Widening of the mediastinum is commonly identified in cases of inhalation anthrax. Pleural effusion and parenchymal infiltrations are also observed. Patients often experience hypothermia and shock, which can lead to death. Up to 50% of patients may develop meningitis as a complication. Inhalation anthrax resembles community-acquired pneumonia and other pulmonary diseases. Recently, progress has been made in inhalation anthrax treatment [65,73]. The most promising treatments include new antibiotics, peptides and bacteriophages enzymes, monoclonal antibodies, protective antigen mutants, and human inter-alpha-inhibitor proteins (IαIp), which are the endogenous plasma proteins identified in humans and function as serine protease inhibitors [74,75].

### 5.4. Injectional Anthrax

This term describes a new clinical form of anthrax wherein the infection occurs in the soft tissue at the injection site, leading to toxemia and sepsis [76]. Conditions such as gas gangrene, necrotizing soft tissue infections, and severe cellulitis should be ruled out [65,69]. Several cases of contaminated heroin-related injection abscesses are recorded among European drug users [77,78].

### 5.5. Meningitis Anthrax

Anthrax meningitis can arise from the bloodborne spread of any clinical form of anthrax, or it can occur alone [79]. The condition should be considered in patients with anthrax who present with severe headaches, changes in mental status (such as confusion), signs of meningitis, or any neurological deficits. About two-thirds of patients with anthrax meningitis experience intracranial bleeding [80]. The majority of anthrax meningitis cases result in death [79,81].

It is not a coincidence that humans serve as unintentional hosts in this livestock–human interaction. For anthrax to be ruled out in cattle and humans who prepare or consume animal products, a thorough investigation must be carried out. All forms of anthrax can cause septicemia and mortality, so it is critical to have immediate laboratory confirmation, medical care, and containments [72,82].

To treat anthrax, broad-spectrum antibiotics like amoxicillin and ciprofloxacin are advised [83]. A systematic review highlighted that most *B. anthracis* isolates are susceptible to current first-line antimicrobials recommended for postexposure prophylaxis and treatment [84]. Nonetheless, the risk of antibiotic-resistant strains always exists [83].

Furthermore, the diagnosis of anthrax is delayed if there are no identifiable symptoms during early infection, resulting in increased levels of anthrax toxin components that could be lethal. If untreated, the condition advances to dyspnea, shock, and collapse, eventually leading to fulminant sepsis and mortality [85].

Anthrax is enzootic and endemic in agricultural regions of sub-Saharan Africa, Central and South America, central and southwestern Asia, and southern and eastern Europe. It is enzootic but not endemic in the United States, Canada, and Western Europe [79]. Anthrax poses a public health threat in the regions of central Asia, the Middle East, Africa, and South America. Around 20,000 to 100,000 human anthrax cases are reported globally every year, and the majority of cases are from rural or poor areas [24].

Between 2010 and 2014 in Odisha, India, 325 human anthrax cases were recorded, with most patients suffering from cutaneous illness [86]. In 2015, 81 human anthrax cases with a 4% mortality rate were reported in Odisha [85]. Similarly, in 2021, an outbreak in five villages of Odisha led to 47 clinically suspected anthrax cases [87]. From 2000 to 2010, 14 out of 30 districts in the Odisha state experienced anthrax outbreaks 61 times, affecting at least 1208 people, with 436 fatalities [88]. The true burden of anthrax in India remains unclear as many cases go unreported, and only a fraction of human cases receive medical attention. Between 1955 and 2014, 120,111 confirmed human cases of anthrax were reported in China, with 4341 fatalities [89]. A review of 426 recorded anthrax cases in Turkey between 1990 and 2007 revealed that 96.9% were cutaneous anthrax, less than 2% were gastrointestinal anthrax, and less than 2% were meningitis anthrax [90].

Human anthrax instances have also been documented in several other countries such as Bhutan [50], Kenya [15], Turkey [35], and Romania [91]. In the USA, in 2001, intentionally contaminated letters with *B. anthracis* spores resulted in 22 anthrax cases (bioterrorism). Since the 2001 anthrax attacks, the Centers for Disease Control and Prevention (CDC) has reported six additional anthrax cases. These include one case of cutaneous anthrax from direct exposure to livestock, one gastrointestinal case from exposure to contaminated animal hides used for drum making, and two more cases each of inhalation and cutaneous anthrax [92]. Anthrax remains rare in humans within the European Union, with only a few cases reported annually. In 2021, there were four confirmed anthrax cases: one in Bulgaria and three in Spain. Additionally, France reported one probable case, and Spain reported two. Out of the 30 European countries that provided data, 27 reported no cases at all [93]. Similarly, anthrax is uncommon in Australia, with only three human cases reported since 2001 [63].

## 6. One Health Approach to Control Anthrax

The concept of “One Health” (OH) emphasizes the interconnectedness of animal, human, and ecosystem health. It advocates for a collaborative, transdisciplinary approach to achieve optimal health outcomes by recognizing that the health of people is closely linked to the health of animals and our shared environment [94]. This approach seeks to integrate efforts across multiple sectors and disciplines to address complex health challenges, particularly those involving zoonotic diseases.

Anthrax, despite being an ancient disease, remains endemic and enzootic in many parts of the world. This persistence is due to several factors, including inadequate public health services, ineffective policies, insufficient research, and poor coordination in addressing sporadic outbreaks [95]. In regions heavily dependent on livestock, conflicts over carcass disposal methods often arise due to the proximity between humans and animals [94]. Improperly disposed animal corpses can end up in rivers, posing significant transmission risks to nearby human and animal communities [96]. The disease’s fragmented surveillance, identification, transmission, diagnosis, and control strategies further complicate efforts to manage and mitigate its impact [97].

The OH approach offers a comprehensive solution to the challenges posed by anthrax. By fostering collaboration across various disciplines and sectors, OH can address the multifaceted nature of anthrax transmission and control. This approach extends beyond the traditional focus on animal–human health connections to include eco-health, agro-ecosystem health, resilience, adaptive management, and sustainability studies [98]. Successful management of human anthrax epidemics, as demonstrated during the 2016 outbreak among the Nenet indigenous peoples in Russia, underscores the importance of coordinated responses and the need for a holistic perspective [99,100]. Investing in OH strategies that promote interdisciplinary collaboration is crucial to breaking the cycle of anthrax and other zoonotic diseases [101]. By integrating input from policymakers and directing research toward practical and politically feasible solutions, the OH approach can address the root causes of zoonotic disease transmission and improve overall health outcomes [102]. Implementing the OH approach to combat anthrax involves several key steps which are discussed below.

### 6.1. Enhanced Surveillance and Monitoring

Establishing robust surveillance systems in high-risk areas is essential to monitor the spread of anthrax and identify outbreaks early [101]. This process involves deploying advanced diagnostic tools and techniques to detect anthrax spores in the environment and animals before they cause human infections. Furthermore, developing integrated data platforms that enable real-time sharing of surveillance data among health agencies, veterinary services, and environmental monitoring organizations is crucial [103]. Such integration allows for the prompt identification of emerging threats and coordinated response efforts. Additionally, providing training and capacity-building programs for health professionals, veterinarians, and environmental scientists is vital to enhance their ability to detect and respond to anthrax outbreaks [104]. Continuous education and simulation exercises can significantly improve readiness and response capabilities.

A study effectively identified high-risk areas for anthrax and highlighted the anthrax persistence in Ha Giang province, Vietnam, though it was constrained by the absence of livestock anthrax and vaccination data before 2010. The introduction of “One Health Circular 16” in 2013 improved collaborative surveillance, leading to better data sharing between human and animal health sectors [105].

### 6.2. Proper Carcass Disposal

Promoting safe carcass disposal methods, such as incineration or deep burial, is essential to prevent environmental contamination and reduce transmission risks [106]. Infected carcasses should be immediately burned or buried at least 6 feet deep without using lime, as recent research suggests that lime can promote the survival of anthrax spores. Lime-treated soil may later release spores through plowing, erosion, scavenger activity, or water contamination, increasing the risk of future outbreaks [107]. Establishing guidelines ensures that carcasses are disposed of in a manner that minimizes the risk of spreading anthrax spores [108]. Additionally, engaging local communities in safe disposal practices is crucial. Raising awareness about the dangers of improper carcass handling and involving communities in implementing disposal protocols can enhance compliance and effectiveness. Community-based monitoring and reporting systems further support these efforts. Moreover, developing and enforcing regulatory frameworks that mandate the proper disposal of animal carcasses, with penalties for non-compliance, is necessary. These frameworks should be backed by infrastructure investments to provide accessible and affordable disposal options, ensuring the comprehensive and effective management of carcass disposal.

### 6.3. Vaccination and Quarantine

Implementing vaccination programs for livestock is crucial for creating herd immunity and preventing the spread of anthrax among animal populations. Maintaining regular vaccination schedules, especially in endemic regions, is essential for effective control [109]. Monitoring the efficacy of vaccines through field studies and laboratory tests ensures that they provide adequate protection against local strains of *B. anthracis*. Based on these findings, the continuous monitoring of their effectiveness and adjustments to vaccination strategies should be undertaken to enhance their effectiveness. As per the guidelines of the WHO, the most effective method for controlling anthrax in domestic livestock is vaccination with the Sterne vaccine, which is inexpensive and highly effective but needs to be repeated annually [23]. In Russia, the live spore vaccine (STI) has been used in a two-dose schedule [110].

Controlling animal infections through vaccination can minimize the incidence of disease in humans. Live spore vaccines for human use and cell-free vaccines containing protective antigens are available in various countries for veterinarians, laboratory workers, and others who are likely to be exposed to anthrax [72]. However, vaccinating the large human population against anthrax is impractical [111]. The risks of vaccine failures and various reactions in vaccinated animals raise concerns about the efficacy of the vaccines among livestock owners [112]. The application of vaccination among some susceptible wildlife seems largely inapplicable, expensive, and irrelevant [25]. Moreover, administering booster doses to livestock and wild animals every year is tardy and costly. Therefore, the vaccination program should be well-organized, coherent with all authorities at all levels of vaccination, well-recorded, and well-reported, rather than randomized and individualized, for the effective utilization of resources and better outcomes. Enhancing veterinary extension services and decentralizing cold chain facilities are also essential for better access to the anthrax vaccine [113].

In addition to vaccination, enforcing quarantine measures to isolate infected animals is necessary to prevent the disease from spreading to other livestock and humans [114]. Clearly defined and effectively communicated quarantine protocols should be provided to farmers and animal handlers. Quarantine should be enforced on flocks and herds affected by anthrax, restricting contact between infected and non-exposed groups and preventing susceptible animals from accessing contaminated areas [115].

The primary control measure for animal anthrax is annual preventive vaccination; however, once an outbreak occurs, other control measures include ring vaccination, proper carcass disposal to avoid further environmental contamination, and quarantine (i.e., limit animal movement from the affected and nearby properties, animal contact with anthrax-contaminated sites, and contact between affected and nonaffected herds) [66].

### 6.4. Public Awareness and Education

Research conducted in different countries has shown a low level of knowledge or awareness, attitude, and preventive practice of anthrax [113,116,117,118]. Thus, raising awareness through educational campaigns about the severity of anthrax, its transmission pathways, and preventive measures is essential. These campaigns should specifically target farmers, animal handlers, healthcare workers, and the general public who are at high risk of anthrax transmission or part of the anthrax transmission cycle. Encouraging community-led initiatives that empower marginalized communities to take proactive measures in preventing and controlling anthrax is also vital [113]. Programs like the African Program for Onchocerciasis Control (APOC) and Community-Led Total Sanitation (CLTS) can serve as models for similar efforts [119,120]. Additionally, utilizing various media platforms, including social media, radio, television, and print, to disseminate information about public health problems like anthrax prevention and control can significantly enhance public knowledge [121,122]. Engaging local influencers and community leaders in these efforts can further extend the reach and impact of the messages, ensuring comprehensive community engagement and awareness. Furthermore, it is crucial to engage with the impacted communities and collaborate across various disciplines to effectively combat poverty, enhance veterinary services, and address traditional meat consumption practices within these communities [113].

A study in Odisha, India, showed a notable improvement in community knowledge and practices regarding anthrax after the implementation of intervention packages based on the OH approach. It highlighted the need for OH interventions to align with health policies, establish robust surveillance systems, and enhance community education for better outbreak prevention and control, especially in anthrax-endemic regions [123].

### 6.5. Legislative and Policy Support

In several countries, the ministries associated with humans, animals, and the environment have their sectorial priorities wherein the OH approach has been overlooked or not effectively implemented [124]. The policymakers in the concerned fields have their different strategies for data collection, disease surveillance, and transmission control, but statistics do not reflect the true prevalence of the diseases due to a lack of data sharing, independent resource mobilization, and effective coordination [71]. Developing and enforcing strong legislative frameworks is crucial to support quarantine measures, regular health check-ups for animals, and coordination among different health sectors. These frameworks should provide clear guidelines and allocate resources for effective implementation. Integrating OH principles into national and regional health policies ensures a coordinated approach to zoonotic disease prevention and control. This integration should involve collaboration among ministries of health, agriculture, environment, and other relevant sectors. Additionally, securing funding and resources to support OH initiatives, including research, surveillance, vaccination, and public education, is essential [125]. Contributions from international organizations, governments, and private sector partners can significantly bolster these efforts.

### 6.6. Cross-Sector Collaboration

Forming interdisciplinary teams that include medical, veterinary, environmental, political, societal, and ecological experts is essential for developing and implementing comprehensive strategies for anthrax control [15]. These teams should meet regularly to share information, coordinate activities, and evaluate progress. Promoting collaborative research projects that explore the interactions between human, animal, and environmental health in the context of anthrax can provide valuable insights into effective prevention and control measures [126]. Additionally, encouraging international cooperation and knowledge exchange is crucial for tackling anthrax and other zoonotic diseases. Countries can significantly benefit from sharing best practices, research findings, and technological innovations.

**Figure 4:**
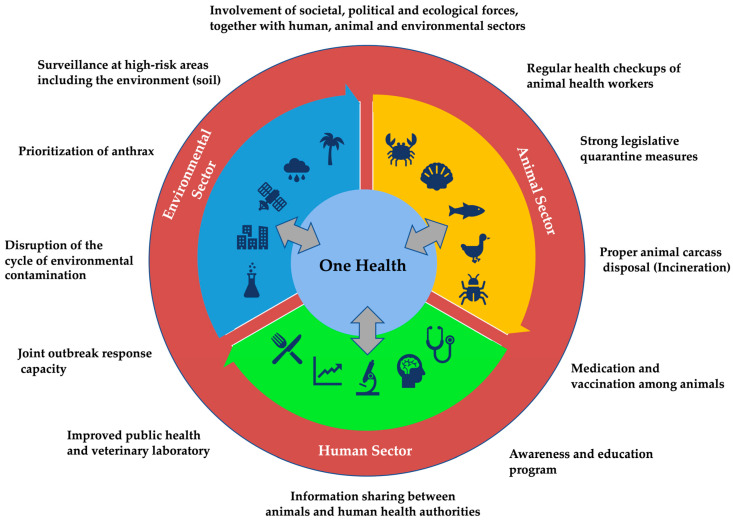
Model framework for collaborative, coordinated, and interdisciplinary One Health approach to control anthrax.

As global health challenges continue to evolve, the OH approach provides a sustainable and adaptive framework for addressing the interconnected health of humans, animals, and the environment (Figure 4). By fostering collaboration, enhancing surveillance, and promoting effective public health policies, the OH approach can significantly reduce the impact of zoonotic diseases and improve health outcomes worldwide. Investing in OH not only addresses current health threats but also prepares us to respond more effectively to emerging health challenges in the future.

Since 2006, CDC Kenya has collaborated with Kenyan government institutions to establish a sustainable OH program, catalyzed by emerging zoonotic threats. This has led to the creation of a cross-sectoral coordinating unit, improved surveillance systems, a trained workforce, and a robust public health scientific program [127].

In developing countries, challenges persist in ensuring the sustainability of the OH program, improving veterinary laboratory diagnostics, and securing the necessary resources to implement more comprehensive control and prevention strategies for zoonotic diseases like anthrax.

## 7. Conclusions

Anthrax, a persistent zoonotic disease caused by *Bacillus anthracis*, poses a significant threat to human, animal, and environmental health. Its resilience and ability to spread across species necessitate a comprehensive One Health approach for successful control and prevention. This review underscores the importance of integrating efforts across medical, veterinary, and environmental sectors to address the complex nature of anthrax transmission. The specific plans for implementing the One Health approach to curb anthrax may differ for different countries depending on various factors, including the extent of disease burden, health infrastructure, environmental and ecological context, intersectoral collaboration, existing One Health framework, education and public awareness, and government policies and regulations. However, enhanced surveillance and monitoring, proper carcass disposal, vaccination programs, public awareness, legislative support, and cross-sector collaboration are key strategies to consider for effective anthrax control. By implementing these measures, we can reduce the incidence of anthrax, protect at-risk populations, and mitigate the impact of future outbreaks. A coordinated One Health approach not only addresses the current anthrax threat but also strengthens our ability to combat other zoonotic diseases, promoting overall global health and resilience.

## Data Availability

No new data were created or analyzed in this study. Data sharing is not applicable to this article.
